# Exploring Variation and Predictors of Residential Fine Particulate Matter Infiltration

**DOI:** 10.3390/ijerph7083211

**Published:** 2010-08-16

**Authors:** Nina A. Clark, Ryan W. Allen, Perry Hystad, Lance Wallace, Sharon D. Dell, Richard Foty, Ewa Dabek-Zlotorzynska, Greg Evans, Amanda J. Wheeler

**Affiliations:** 1 Health Canada, 269 Laurier Ave West, Ottawa, Ontario K1A 0K9, Canada; E-Mail: nina.clark@hc-sc.gc.ca (N.A.C.); 2 Simon Fraser University, 8888 University Drive, Burnaby, British Columbia V5A 1S6, Canada; E-Mail: allenr@sfu.ca (R.W.A.); 3 University of British Columbia, 2206 East Mall, Vancouver, British Columbia V6T 1Z3, Canada; E-Mail: phystad@gmail.com (P.H.); 4 11568 Woodhollow Ct, Reston, VA 20191, USA; E-Mail: lwallace73@gmail.com (L.W.); 5 The Hospital for Sick Children, 555 University Avenue, Toronto, Ontario M5G 1X8, Canada; E-Mail: sharon.dell@sickkids.ca (S.D.D.); richard.foty@sickkids.ca (R.F.); 6 Environment Canada, 335 River Road, Ottawa, Ontario K1V 1C7, Canada; E-Mail: Ewa.Dabek@ec.gc.ca (E.D.-Z.); 7 University of Toronto, 200 College Street, Toronto, Ontario M5S 3E5, Canada; E-Mail: greg.evans@utoronto.ca (G.E)

**Keywords:** air exchange, air quality, indoor, infiltration, fine particulate matter, PM_2.5_, residential, sulphur

## Abstract

Although individuals spend the majority of their time indoors, most epidemiological studies estimate personal air pollution exposures based on outdoor levels. This almost certainly results in exposure misclassification as pollutant infiltration varies between homes. However, it is often not possible to collect detailed measures of infiltration for individual homes in large-scale epidemiological studies and thus there is currently a need to develop models that can be used to predict these values. To address this need, we examined infiltration of fine particulate matter (PM_2.5_) and identified determinants of infiltration for 46 residential homes in Toronto, Canada. Infiltration was estimated using the indoor/outdoor sulphur ratio and information on hypothesized predictors of infiltration were collected using questionnaires and publicly available databases. Multiple linear regression was used to develop the models. Mean infiltration was 0.52 ± 0.21 with no significant difference across heating and non-heating seasons. Predictors of infiltration were air exchange, presence of central air conditioning, and forced air heating. These variables accounted for 38% of the variability in infiltration. Without air exchange, the model accounted for 26% of the variability. Effective modelling of infiltration in individual homes remains difficult, although key variables such as use of central air conditioning show potential as an easily attainable indicator of infiltration.

## 1. Introduction

Exposure to outdoor particulate matter (PM) has been linked to a wide variety of health effects [1–3]. However, exposure assessment in epidemiological studies remains a challenge and misclassification limits the power of many studies to find true effects [4,5]. A nearly universal source of exposure misclassification in studies of outdoor air pollution results from estimating exposure based on outdoor levels alone. Indeed, individuals’ true exposures are determined by their time-activity patterns and levels of pollutants in each microenvironment where they spend time—typically indoors at home [6,7]. Surveys of activity patterns for Canada and the United States suggest that adults spend an average of about 88% of their time indoors, 66% of which is in their homes [8–10]. Exposure misclassification occurs because infiltration of outdoor pollution can vary significantly between residences and also within residences across time [11–13].

There are important differences between indoor and outdoor PM composition in addition to differences in concentration. Outdoor generated PM contains relatively more sulphates, nitrates, strong acids, and toxic metals as compared with PM generated in homes, which contains more house dust, endotoxins, mold spores, and fresh combustion products [9]. Consequently, indoor and outdoor generated PM appear to have different health effects. Health effects of indoor PM_2.5_ have not been thoroughly studied, but have been linked to respiratory problems [14–16]. Several studies that measured exposure to both indoor and outdoor PM_2.5_ have found that outdoor-generated PM generally showed stronger relationships with respiratory [17–19] and cardiovascular [18] markers than total or indoor-generated PM_2.5_ exposure. Therefore, differences in infiltration of outdoor generated PM_2.5_ across homes could introduce exposure misclassification and may bias health effect estimates [20].

Infiltration differences across homes have the potential to cause differential misclassification, where the degree of misclassification depends on other variables in the analysis (such as disease status). For example, infiltration has been found to depend on characteristics that may also influence disease status, such as socioeconomic status [21,22]. Unlike non-differential misclassification, which generally causes a bias to the null, differential misclassification may cause unexpected effects in health effect estimates by causing a bias toward or away from the null [20].

The infiltration factor (F_inf_) is defined as the equilibrium proportion of outdoor PM that penetrates indoors and remains suspended [23]. This is determined by the particle penetration efficiency (*P*, unitless), particle deposition rate (*k*, h^−1^), and the air exchange rate (*a*, h^−1^) according to the following equation:

(1) $$F_{\text{inf}}=\frac{Pa}{a+k}$$

Various studies have estimated F_inf_ for residential homes. Most commonly they have employed sulphur or sulphate as a tracer of outdoor PM_2.5_ [24–28], although indoor and outdoor measures of PM_2.5_ have also commonly been used to estimate F_inf_ [11,13,21,29–31]. Sulphur has few indoor sources and has been demonstrated to be representative of total outdoor infiltrated PM_2.5_ [9,32,33].

Though a number of studies have estimated F_inf_ in individual homes and some panel studies have considered F_inf_ in epidemiologic analyses [17–19], the need to include both indoor and outdoor pollution measurements to estimate F_inf_ has prevented inclusion of infiltration in large-scale epidemiologic studies. The inclusion of infiltration ‘modification’ factors has been limited to air conditioning variables, such as community-wide prevalence of central air conditioning [34,35]. Given the importance of F_inf_ (studies have shown F_inf_ differences can modify indoor exposure rates by a magnitude of 2–4 [11,21,24]), it is important to further examine how F_inf_ varies between homes and what drives these differences. The goal of this study was to determine household factors that influence F_inf_, as indicated by the sulphur tracer method [27] for homes in Toronto, Ontario.

## 2. Experimental Section

### 2.1. Data Collection

Sixty homes from the Toronto area were recruited for the study. Homes were randomly selected from among 1,500 owner-occupied homes with secure backyards participating in the population-based Toronto Child Health Evaluation Questionnaire (T-CHEQ) study [36]. The T-CHEQ sample as a whole (n = 5,619) is representative of the population in terms of socioeconomic and housing variability as compared to the 2001 Canada census [36], however, the criteria of home-ownership with backyard biased the current study to a somewhat higher socioeconomic status. Fifty homes were initially recruited to participate in the indoor and outdoor sampling in 2006 (August to November) and an additional 10 homes were recruited to participate in 2007 (July).

Participating households completed a baseline questionnaire before sampling began. The questionnaire was a detailed home assessment, including variables that were anticipated to influence F_inf_: home age and size [11,37], home value [21], heating and cooling systems used [21,24], presence of air filters [11,17], presence of storm windows [11,24], and number of residents and pets in the home (which have been observed to increase F_inf_ due to increased air exchange) [37,38]. The assessment was completed by trained technicians along with the home owner during an inspection of the home. Unfortunately, it was not possible to collect information on activities in the home during sampling such as cooking, cleaning, window opening and air conditioner use.

Each home was sampled over a five-day period, with approximately 4 homes sampled concurrently. Most inspections began in the middle of the week and included weekdays and weekend days. Indoor measurements were collected at breathing height inside participants’ homes, typically in the family or living room where participants spent the majority of their time. When possible, measurements were collected away from ventilation ducts, fireplaces, and TVs as these may cause elevated concentrations of particles. Outdoor samplers were located in the backyard of the participants’ homes several meters away from the house and away from any combustion sources such as barbeques, automobiles and other localized outdoor sources of air pollution. The outdoor sampling height was approximately 1.5 meters above ground.

Indoor and outdoor PM_2.5_ mass was sampled over the entire 5-day period using a Chempass Multi-Component Sampling System (Model 3400, R&P/Thermo Scientific, Waltham, MA). The indoor and outdoor systems used a BGI pump with an AC adapter. The sampler target flow rate was 1.8 L/minute and flow rates more than 20% different from the target rate were considered invalid. Flow rates were assessed pre and post sampling using a soap bubble flow meter (AP Buck, Orlando, FL). The samples were collected on 37 mm Teflon™ filters, which were measured gravimetrically following US EPA quality assurance guidelines [39]. Briefly, filters were pre-conditioned for a minimum of 24 h before weighing at a constant air temperature of 21 ^o^C (±0.5 ^o^C) and constant relative humidity (RH) of 40% (±1%).

All samples were analyzed for total sulphur by a Panalytical Epilson 5 energy dispersive x-ray fluorescence (ED-XRF) spectrometer with a 600-watt gadolinium target tube as an excitation source. Micromatter® standard reference samples were used for the quantitative calibration of the system. A calibration check was performed by analyzing NIST standards (SRM1832 and SRM1833). Following the measurement process, data treatment was performed using complex algorithms that corrected for background signal, spectral interferences, and X-ray signal loss due to absorption by the sample mass. Blank filters were used for background subtraction. Typical analytical limits of detection and uncertainty were estimated to be 3.0 ng/cm^2^ and 5%, respectively.

The indoor temperature and relative humidity were measured using HOBO Temperature Relative Humidity Data Logger (U10-003, Onset Corporation, Bourne, MA, USA) at three-minute time intervals. Outdoor meteorological data were obtained from the National Climate Data and Information Archive of Environment Canada [40].

Air exchange rate (AER) (the rate at which outdoor air replaces indoor air) was measured in the 50 homes monitored in 2006 using a perfluorocarbon tracer (PFT) source emitting a tracer at a constant rate. This was captured by a capillary adsorption tube (CAT), a passive sampler containing a small amount of activated charcoal adsorbing material [41]. Four PFTs were placed in the corners of the floor where the indoor equipment was located, and one CAT was placed near the center of the floor at head height. After exposure, the CAT was shipped to the lab and analyzed by gas chromatography with an electron capture detector (GC/ECD). Air exchange rate was then calculated using the concentration of PFT detected on the CAT and the volume of the home (estimated by multiplying square footage of the home by the technician’s estimate of ceiling height for the home). Air exchange was measured on the main living floor (in family or living room) only as this has been demonstrated to be a relatively accurate estimate of air exchange for the house: Wallace *et al.* [42] found inter-floor differences of less than 10% in a three-story home measured over a year. However, it was not possible to assess factors that may influence mixing of air in the home, such as closed doors. Closed doors to rooms or whole floors of the home for extended periods of time (e.g., nighttime) would reduce the effective volume of the home and thereby cause an underestimate of air exchange.

In addition to information collected during exposure monitoring, other variables of interest were collected from publicly available tax records [43] and geographic information system (GIS) data. Specifically, information was obtained on home size and market value from the Municipal Property Assessment Corporation (MPAC) of Ontario, as these factors have been observed to influence F_inf_ [21,37]. GIS data were used to determine the distance of each home to the nearest highway. Roadway classifications in the CanMap RouteLogistics dataset were used, including highways and expressways (400 series highways and expressways).

### 2.2. Statistical Methods

Infiltration was calculated using the ratio of the indoor concentration and the outdoor concentration of sulphur (Equation 2).

(2) $$F_{\text{inf}}^{S}=\frac{S_{\text{in}}}{S_{\text{out}}}$$

Linear regression was used to examine the association between housing and climatic variables and F_inf_. Right-skewed variables were log-normally transformed for regression modelling. Ambient temperature was examined as an effect modifier due to its influence on window opening behaviour, use of air conditioners and indoor/outdoor temperature differences. This was included as an interaction term indicating whether outdoor temperature was above or below 18 °C at time of monitoring. Many of the housing and climate characteristics are expected to be correlated and thus bivariate regressions may only indicate an indirect effect. However, variables without a direct effect may still be helpful for predicting F_inf_ in studies with limited data, and are therefore presented along with multiple linear regression models.

To understand the best predictors of F_inf_, variables that were associated with F_inf_ (p < 0.10) in a simple linear regression were entered into multiple linear regression models. Two sets of models were examined: one set in which air exchange rate was a potential predictor variable and another set in which it was not, as this variable is often not available in larger-scale epidemiology studies. A stepwise procedure was used to select the most appropriate models, with p < 0.10 as the cut-off for inclusion of variables. The guideline of a minimum of 10 observations per degree of freedom was followed to guard against over-fitting. Collinearity between variables was examined using Pearson and Spearman correlation coefficients and t-tests where appropriate, as well as using the “collin” option available for the REG procedure in SAS 9.1 (SAS Institute Inc., Cary, NC, USA).

## 3. Results

Table 1 provides descriptive data for F_inf_, pollutant measurements, and home and climate characteristics that were hypothesized *a priori* to have an effect on F_inf_. Of the 60 participating homes, 53 were analyzed for sulphur content indoors and outdoors. Of the calculated F_inf_ values, two homes had an indoor/outdoor sulphur ratio greater than one (2.89 and 3.09) and likely indicate an undetected indoor source of sulphur, for example, an unreported humidifier in the home [9]. Only values of F_inf_ between 0 and 1 are physically valid; therefore, these two homes were excluded from further statistical analysis. An additional five homes had a sulphur ratio >0.90 (ranging from 0.93–0.98) without a correspondingly high air exchange rate; these homes were also excluded from further analysis, leaving a total of 46 homes for analysis. As compared with included homes, the excluded homes were more likely to have higher levels of indoor PM_2.5_ (13.9 ±7.08 μg/m^3^ *vs.* 8.17 ± 5.18 μg/m^3^, p-value = 0.01) and indoor sulphur (1.08 ±0.35 μg/m^3^ *vs.* 0.46 ± 0.31 μg/m^3^, p < 0.001) (consistent with having an indoor source). They did not differ with respect to other analyzed variables. Additional homes were excluded from specific analyses when covariate data was not available; this was most significant for air exchange which was not measured in the 10 homes sampled in 2007. The participating home-owners were of higher socioeconomic status as compared with the general Canadian population, but home values were representative of the Greater Toronto Area, where the average single-family home price is $435,064 [44].

The mean (±standard deviation) F_inf_ among the 46 included homes was 0.52 ± 0.21, ranging from 0.08 to 0.88 with approximately normal distribution. Means were not significantly different based on whether homes were sampled during times when heating might occur: when the outdoor temperature was below 18 °C, the average F_inf_ was 0.54 ± 0.22 as compared with 0.48 ± 0.21 when temperature was above 18 °C. This difference was not statistically significant (p = 0.34).

The mean sulphur concentration was 0.46 ± 0.31 μg/m^3^ indoors and 0.76 ± 0.36 μg/m^3^ outdoors. On average, PM_2.5_ concentrations were slightly higher outdoors (9.72 ± 3.90 μg/m^3^) than indoors (8.17 ± 5.18 μg/m^3^).

The results of simple linear regression are presented in Table 2. Since the great majority of homes had air conditioners (96%), a smaller group of “regular users of air conditioning” was also defined: homes that reported using their air conditioner at least 30 days/year. This group consisted of 24 homes (52%). No other information on use of heating or cooling systems was available, including whether they were actually used during sampling.

In multiple linear regression modeling, air exchange rate, use of air conditioning more than 30 days/year, and having forced air heating remained as independent predictors of F_inf_, predicting 38% of variability (Table 3). Without air exchange, the model was able to predict 26% of variability.

## 4. Discussion

Infiltration is rarely measured in studies of air pollution and health because it is generally not feasible to do so for a large number of homes. However, it is an important determinant of personal exposures to outdoor air pollution. Variability between and within homes can be significant, for example, Allen *et al.* [11] and Wallace *et al.* [27] found approximately a three- to four-fold range in F_inf_ estimates between individual homes, ranging from 0.24 to 1.00 and 0.26 to 0.87, respectively. Our estimates of F_inf_ show a ten-fold difference across homes, ranging from 0.08 to 0.88. Simple measures of PM_2.5_ concentrations inside the home are insufficient to address these differences in exposure, since recent work suggest that outdoor particulate matter may be more detrimental to health than particulate matter generated in homes [9,18]. However, activities in homes such as cooking have also been found to contribute significant amounts of potentially toxic PM [45]. Development of reliable and efficient methods for modelling infiltration among large populations is therefore an important goal for improving air pollution health studies.

Toronto experiences relatively hot summers (average daily highs of 27 °C in July) and cold winters (average daily lows of −11 °C in January) [46]. These temperatures necessitate ensuring that buildings are well sealed from outdoor air and typically homes in Canada use mechanical heating/cooling for occupant comfort. It is therefore expected that Toronto homes would have relatively low infiltration as compared with homes in more moderate climates. Indeed, the mean infiltration estimate of 0.52 for homes in Toronto is lower than generally observed in milder climates, such as 0.65 in Seattle [11], 0.62 in Victoria, BC [21] (both averaged over heating and non-heating seasons) and 0.70 in Riverside, CA (measured in fall) [29]. Regions with similar temperature extremes to Toronto that have also conducted measurements across heating and non-heating seasons have found similar estimates: 0.45 and 0.55 in North Carolina [27,31]; and 0.59 in Helsinki [37].

Another seasonally important source of unexplained variability is likely to be window opening behaviour. Meng *et al.* [47] observed that infiltration was highest when outdoor temperatures were close to 20 °C and decreased at higher and lower temperatures, likely due to window opening. Open windows significantly increase air exchange rates (by an estimated 1.1 air changes per hour as measured by Wallace *et al.* 2002 [42]) and thereby also increased F_inf_ [11,42,48].

Regular use of air conditioning was a consistent predictor of infiltration, despite a lack of information on actual use during sampling. Homes that reported using air conditioning on more than 30 days per year had significantly lower infiltration than homes that either did not have air conditioning or used it less than 30 days per year. Air conditioned homes are expected to have lower infiltration due to being more tightly sealed as well as experiencing deposition resulting from the ventilation system. This has been demonstrated in a number of studies including Dockery and Spengler [24], Suh *et al.* [25], Long *et al.* [49], and Meng *et al.* [12,47]. In addition, epidemiological studies have found that air conditioning prevalence accounts for some of the variability observed in the health effects of particulate matter across different cities. Janssen *et al.* [35] and Medina-Ramon *et al.* [50] found that across cities in the US, higher prevalence of central air conditioning attenuated the acute effects of PM_10_. A meta-analysis of 19 studies of particulate matter in the US also found that mortality was modified by central air conditioning prevalence: mortality increased by 0.57% (95% CI: 0.39–0.74%) per 10 μg/m^3^ increase in ambient PM_10_ in communities with central air conditioning prevalence greater than 30% *vs.* 0.76% (0.54–0.98%) with prevalence less than 30% [51]. A recent study by Bell *et al.* [52] confirmed the findings of attenuating effects of central air conditioning use on cardiovascular and respiratory diseases as well as mortality. Differences in central air conditioning prevalence across communities accounted for 17% of the variability in cardiovascular hospitalizations.

Publicly available property assessment data from MPAC was of limited utility in predicting infiltration in these Toronto homes. Hystad *et al.* [21] also examined the usefulness of property assessment data in Seattle, WA and Victoria, BC for predicting infiltration and found that the value of the structure, also known as improved value, served as an effective surrogate for a number of other housing variables and predicted F_inf_. Homes with structure value below the median had 15% higher infiltration than homes above the median. However, total market value did not predict F_inf_ in Seattle, nor did it in Toronto. Unlike data sources in BC and WA, MPAC does not separate the home value into structure value and land value, preventing further examination of this relationship.

Of the other variables obtained from MPAC, only presence of forced air heating predicted infiltration, accounting for 7% of the variability. Although presence of central air conditioning was available through MPAC, comparison with home owner report suggests that this variable is not well maintained in the database; of the 39 homes who reported having central air conditioning, only 24 were classified as such in the MPAC database. Therefore, questionnaire data was relied on to classify homes for air conditioning presence.

Home age has previously been found to influence infiltration, though inconsistently across different studies. This is not unexpected as different building methods influencing tightness of the homes differ across regions and over time, and may be altered by home renovations (such as updated windows and insulation). There was a significant linear relationship with older homes having higher infiltration, although this relationship did not remain significant in multivariate models. A trend of lower infiltration in newer homes was also observed by Lachenmyer and Hidy [30], Hanninen *et al.* [37], and Hystad *et al.* [21], while Allen *et al.* [11] found that older homes had lower infiltration in a sample of Seattle homes. No effect of home age was observed by Meng *et al.* [47].

Distance to expressways was not associated with F_inf_. It was hypothesized that homes near busy roads may have lower infiltration due to windows being closed to reduce noise, however it is important to note that only 5 (11%) of study homes were within 500 m of expressways and that previous research has shown simple distance-based metrics to be only moderate predictors of noise [53].

## 5. Conclusions

This study, as well as others, has found that a large portion of the variability in infiltration cannot be explained using housing and climate characteristics. While information on air conditioning use and outdoor temperature can serve as proxy measures of window opening, they do not capture many important factors, such as the number of open windows, the size of the opening(s), and the duration that windows are left open, which could influence infiltrating particles. These variables were unavailable for this study to be able to identify their contribution.

F_inf_ estimates found in Toronto homes are comparable to previous estimates from similar climates and homes and confirm that typically there is a high degree of variation in infiltration across different homes. Exposure to outdoor pollution can be markedly different even for individuals living in close proximity and experiencing similar outdoor levels. Effective modelling of F_inf_ in individual homes remains difficult, although key variables such as use of central air conditioning show potential as an easily attainable indicator of F_inf_. These are important considerations when investigating the health effects of air pollution in large epidemiological studies especially when considering providing guidance on high air pollution days to vulnerable populations.

## Figures and Tables

**Table 1.** Baseline and measured characteristics of 46 homes in Toronto. For household characteristics that were not obtained via questionnaire, the data source is indicated in brackets.

**Measurement Characteristics**	**Total N**	**Mean (standard deviation)**
Finf	46	0.52 ± 0.21
PM_2.5_ indoors	46	8.17 ± 5.18 μg/m^3^
PM_2.5_ outdoors	46	9.72 ± 3.90 μg/m^3^
Sulphur indoors	46	0.46 ± 0.31 μg/m^3^
Sulphur outdoors	46	0.76 ± 0.36 μg/m^3^
Air exchange	35	0.22 ± 0.15/h
Indoor temperature	46	22.0 ± 2.1 °C
Indoor relative humidity	46	52.0 ± 7.5 %
Outdoor temperature	46	14.6 ± 6.2 °C
Outdoor relative humidity	46	72.6 ±7.7%
**Household Characteristics**		**Median (quartile range)**
Number of people in the home	44	4 (4–5)
Year home built (MPAC)	44	1948 (1925–1967)
Market value of home (MPAC)	44	$437,000 ($330,000–558,000)
Distance to expressway (GIS)	46	1.85 km (1.3–2.6 km)
		**Frequency (percentage)**
Forced air heating (MPAC)	46	33 (72%)
Have air conditioner (central or window unit)	46	44 (96%)
Have central air conditioner	46	39 (85%)
Use air conditioning > 30 days/year	46	24 (52%)
Wood burning fireplace	46	20 (43%)
Air cleaning filter on furnace	45	34 (76%)
Premium air cleaning filter on furnace	46	16 (36%)
Dog or cat in the home	46	17 (37%)
Storm windows	46	10 (22%)

**Table 2.** Simple linear regression of infiltration (F_inf_) with housing and climate characteristics predicted to influence F_inf_. Bold indicates regression with a p-value under 0.10.

Independent Variable	N	Regression Coefficient	p-value	Standard Error	R^2^
**Ln of Air exchange**	**35**	**0.139**	**0.003**	**0.043**	**0.24**
Absolute temperature difference between indoors and outdoors (°C)	46	−0.003	0.699	0.007	0.00
Number of people in the home	44	0.003	0.933	0.037	0.00
**Year home built**	**44**	**−0.003**	**0.011**	**0.001**	**0.14**
Market value of home ($100,000)	44	−0.002	0.849	0.012	0.00
Distance to expressway (km)	46	0.023	0.381	0.026	0.02
**Use air conditioning >30 days/year**	**46**	**−0.201**	**0.001**	**0.056**	**0.23**
**Forced air heating (0/1)**	**46**	**−0.130**	**0.078**	**0.072**	**0.07**
**Wood burning fireplace (0/1)**	**46**	**0.120**	**0.056**	**0.061**	**0.08**
Air cleaning filter on furnace (0/1)	45	−0.097	0.198	0.074	0.04
Premium filter on furnace (0/1)	45	0.083	0.218	0.067	0.04
Dog or cat in the home (0/1)	46	0.029	0.656	0.066	0.00
Storm windows (0/1)	46	0.121	0.113	0.075	0.06

**Table 3.** Multivariate models predicting infiltration factor (F_inf_) using climate and housing characteristics.

**Independent Variables**	**Model N^a^**	**Regression Coefficient**	**Standard Error**	**P-value**	**Adjusted Model R^2^**
Including air exchange as potential predictor					
1 Intercept Ln of air exchange Use air conditioning > 30 d/yr Forced air heating (0/1)	35	0.870 0.114 −0.144 −0.103	0.082 0.039 0.055 0.058	<0.0001 0.01 0.01 0.08	38%
Excluding air exchange as potential predictor					
2 Intercept Use air conditioning > 30 d/yr Forced air heating (0/1)	35	0.698 −0.179 −0.107	0.062 0.060 0.064	<0.0001 0.01 0.10	23%
3 Intercept Use air conditioning > 30 d/yr Forced air heating (0/1)	46	0.708 −0.193 −0.120	0.057 0.054 0.060	<0.0001 0.00 0.05	26%

a Models 1 and 2 are performed on the same homes for direct comparability, while Model 3 includes additional homes for which air exchange rate was not available.
